# Community Factors Shaping Early Age at First Sex among Adolescents in Burkina Faso, Ghana, Malawi, and Uganda

**Published:** 2014-06

**Authors:** Rob Stephenson, Calleen Simon, Catherine Finneran

**Affiliations:** Hubert Department of Global Health, Rollins School of Public Health, Emory, Atlanta, GA, USA

**Keywords:** Adolescents, Community, Multiple partnerships, Sexual behaviour, Sexual debut, Youth, Africa

## Abstract

Using data from the National Survey of Adolescents (2004), we examine the community-level factors associated with early age at first sex among adolescents 14-19 years old in four African countries. Regression models are fitted separately by sex for each country for an outcome measuring early age at first sex, with a focus on community-level factors as potential influences of age on sexual debut. The community-level factors associated with adolescents’ sexual debut vary widely by both country and gender. Community influences that emerge as risk or protective factors of early sexual debut include community levels of adolescent marriage, wealth, religious group affiliation, sex education, parental monitoring, reproductive health knowledge, media exposure, membership in adolescent social group, and use of alcohol. Results indicate the importance of context-specific understanding of adolescents’ sexual behaviour and suggest how elements of place should be harnessed in the development of effective HIV and sexual health interventions.

## INTRODUCTION

In sub-Saharan Africa, nearly 60% of young women and 45% of young men have had sex before the age of 18 years. There is a widening gap between initiation of sexual act and marriage, resulting in a longer period of pre-marital sexual activity (1,2). Early initiation of sexual act and an increased period of sexual activity before marriage can lead to increased risk of both HIV seroconversion and unintended pregnancies among the youths, the sequelae of which are already observed in the literature on epidemiology. In 2007, 45% of all HIV infections occurred among the youths aged 15-25 years (3). Worldwide, an estimated 12-14 million adolescent pregnancies occur each year in the developing world, and in sub-Saharan Africa, between 10% and 79% of all births to women below 20 years of age are reported to be mistimed or unwanted (4).

The importance of preventing HIV and unintended pregnancies among young people has led to a plethora of research examining individual-level factors associated with early initiation of sexual act (5-10), i.e. the existing literature tends to focus on an individual's own characteristics (e.g. religion, ethnicity, and educational level) when seeking to determine risks of adverse sexual health outcomes. Relatively few analyses, however, have gone beyond such individual-level factors to explore how community-level factors may be associated with sexual behaviour, knowledge, and perceptions among young people (11-20). This paper examines such community-level, contextual influences on the early initiation of sexual behaviour among adolescents in Burkina Faso, Ghana, Malawi, and Uganda. A fuller understanding of how the context in which the youths live influences their sexual behaviour has the potential to inform both HIV and unintended pregnancy prevention programmes.

Individual-level factors associated with early initiation of sexual act in a variety of contexts are relatively well-documented. At the individual level, males (21-25) are more likely to have initiated sexual activity compared to females, as are older youths (1,7,9,10,23,26,27) compared to younger youths. Married female youths have been shown to be more sexually active and less likely to use condoms compared to unmarried female youths but unmarried female youths tend to report having comparatively more sexual partners (28,29). The associations between individual-level education and sexual behaviour are less clear. Being currently enrolled in school is found to be associated with increased sexual activity in some studies (10,22) while other researchers have found that current school enrollment is associated with reduced sexual activity and fewer sexual partners (1,5,9,30-32). Knowledge of reproductive health and HIV/AIDS has generally been shown to be correlated with reduced sexual risk-taking (31), and adolescents who disapprove the premarital sex are less likely to be sexually experienced than adolescents with more permissive views of sex (33). In a clear example of the relationships between community-level factors and individual-level factors, use of alcohol among the youths is associated with an increased likelihood of sexual activity and multiple partners (7,8,25,31), possibly because a young person who drinks alcohol may more often be in social environments with norms that permit consumption of alcohol and/or sexual risk-taking (34,35). However, little empirical work has sought to clarify the exact nature of the relationship between these individual-level factors and the community-level factors (e.g. individual use of alcohol versus community attitudes towards the use of alcohol).

Although less-commonly examined, several notable studies in the literature have begun to examine the influence of household-level factors on the sexual behaviour of the youths, particularly the influence of parental behaviours on the youths’ sexual behaviour. For example, adolescents living in wealthier households are less likely to initiate sex early (10) compared to adolescents living in poorer households. Overall, adolescents who live with both parents, who report communication with their parents about sex and who perceive high levels of parental monitoring are accordingly less likely to report high levels of sexual risk-taking (1,7,9,21,23,31,33,36). However, two studies have shown no association or non-protective associations between parent-adolescent relationships and risky sex (1,9). While an examination of household-level factors expands the scope of inquiry beyond the individuals by critically examining the context in which they live, few studies have expanded this scope of inquiry to the level of the community itself.

Evidence indicates that, despite the wide variation in the characteristics of communities in which young people live, community characteristics also appear to shape the sexual behaviour of the youths (11,16-20). Billy *et al*. (11) suggest that community influences on the youths’ sexual behaviour operate through two distinct yet related pathways: opportunity structures (including paths for future social mobility, availability of reproductive health services for adolescents, and demographic makeup), and normative environments. Paths of future social mobility in a community may influence the way in which adolescents perceive the opportunity costs of sex (e.g. risking an unintended pregnancy and/or acquisition of a sexually transmitted infection) while the availability of reproductive health information and services for adolescents may influence sexual decision-making. The demographic characteristics of the community may help determine the availability of potential sexual partners. The normative environment of a community contributes to the characteristics of all three opportunity structures by, for example, dictating the social and community costs of an unintended, extra-marital pregnancy. Building on the work of Billy *et al.* (11), Stephenson (13-15) has documented that, in addition to shaping the sexual behaviour of adolescents, the community characteristics also have significant impacts on the sexual knowledge and HIV-related stigma of adolescents. Stephenson's findings show that higher community levels of education, community levels of HIV/AIDS-related knowledge, and higher tolerance towards HIV-positive individuals at the community level are associated with decreased risky sex among young people (13-15). These findings are particularly important given that the analyses were drawn from data from three demographically distinct African countries. While Stephenson's previous work focused on the youths aged 15-24 years, few published studies have endeavoured to examine the impact of these community-level characteristics on younger adolescents (i.e. youths as young as 12 years).

Indeed, the existing literature regarding community influences on adolescents’ sexual behaviour have tended to focus on the ways in which peer networks and social groups may influence an adolescent's decision-making around sex. For example, some studies have found no association between belonging to a social group or club and risky sexual behaviour (5) while others indicate both protective (37) and antagonistic (31) associations between social group membership and risky sexual behaviour of adolescents. Peer networks, however, appear to exert greater influence on sexual behaviour of adolescents. Adolescents with a larger number of friends of the opposite sex are more likely to be sexually active whereas adolescents with more friends of the same sex have been shown to be less likely to be sexually active (5). Perceptions of peer sexual behaviour are also predictive of individual behaviour. The youths who believe that their friends are sexually active are more likely to have initiated sex and have more partners compared to the youths who perceived that their friends are not sexually active (9,31,33). Despite this, few studies have examined how community-level characteristics beyond the peer/social group may influence the sexual behaviour of young people.

The analysis presented here builds on our previous understanding of the factors associated with early initiation of sexual act by analyzing the contextual influences on sexual behaviour of adolescents for samples of young adolescents in Burkina Faso, Ghana, Malawi, and Uganda. This study endeavours to add to the literature by considering a variety of community-level influences, several of which are novel in the literature, in addition to considering individual-level factors, such as age, religion, and ethnicity. Additionally, the analysis uses a community-specific measure of early age at first sex, allowing for contextual comparisons of the level of sexual risk across several countries. By making comparisons across both sexes and countries, this study contributes to a broader understanding of the community-level factors associated with risky sexual behaviour and informs community-based programmes to prevent HIV and unintended pregnancy in young people.

## MATERIALS AND METHODS

### Setting

The four countries under study, all of which exhibit extremely young population age-structures, were selected to represent different cultural contexts, HIV prevalence profiles, and tendencies in sexual behaviour of adolescents. Between 26.5% and 62.4% of the young people aged 18-24 years have had sex before the age of 18 years in these four countries (38-41). The fertility rate among adolescents varied, although it was high in all four countries: 64 births per 1,000 women aged 15-19 years in Ghana, 131 in Burkina Faso, 135 in Malawi, and 150 in Uganda (42). Between 20% and 59% of all births to mothers aged less than 20 years in these four countries were mistimed or unwanted (43). Like adolescent fertility rates, HIV prevalence varied across countries as well. HIV prevalence was the highest in Malawi, with a prevalence of 8.4% among females and 2.4% among males aged 15-24 years (3). Among Ugandan youths aged 15-24 years, 3.9% of females and 1.3% of males were HIV-positive (3). Like Uganda and Malawi, more young women than men in both Burkina Faso and Ghana were living with HIV. In Ghana, 1.3% of females and 0.4% of males aged 15-24 years were living with HIV (3). In Burkina Faso, HIV prevalence was 0.9% among young females and 0.5% among young males (3).

### Data

The data used in this study are obtained from the 2004 National Survey of Adolescents. These nationally-representative surveys were carried out as part of the ongoing study “Protecting the Next Generation: Understanding the Sexual and Reproductive Health Needs of Young People,” led by the Guttmacher Institute. The surveys were conducted in 2004 and used a two-stage stratified sampling design. In the first stage, enumeration areas were systematically selected in each country.  Second, the households were randomly selected per enumeration area from a household list. All 12-19 years old adolescents who had spent the night in the household prior to the survey were eligible to be interviewed. Overall response rates ranged from 87% in Uganda to 95% in Burkina Faso. Further details about the survey content and methodology are publicly available (43,44-47). Interviews were completed with the following numbers of persons aged 12 to 19 years: 6,015 in Burkina Faso (2,965 females and 3,050 males), 4,505 in Ghana (2,230 females and 2,275 males), 4,080 in Malawi (2,001 females and 2,079 males), and 5,262 in Uganda (2,678 females and 2,584 males).

### Methods

This analysis focuses on ‘early age at first sex’ as a sexual risk. This outcome was constructed by determining the median age at first sex for each gender and country. Early age at first sex was then defined as reporting an age at first sex below the sex- and country-specific median age. All adolescents younger than that of the median age were excluded from the analysis as those adolescents had not reached an age where they would have been eligible for not having had first sex at that early age. In other words, those adolescents below the median age of the first sex were at risk only for having first sex at early age and not yet at risk of not having first sex at early age. Hence, adolescents below the age of the median age of first sex were not a comparable population to adolescents at or over the median age at first sex. Essentially, adolescents below the median age at first sex in a given country could only be classified as experiencing the outcome but were not eligible to be classified as not experiencing this as determination of whether or not they had experienced first sex at early age is only possible once the respondent reached or passed the median age at first sex. In each country and for each sexual act, the median age of first sex varied, thus the age-range of the youths included in the analysis of this outcome also varied in each country and gender [Burkina Faso: females aged 15-19 years, n=1,684, males aged 15-19 years, n=1,704; Ghana: females aged 16-19 years, n=991, males aged 15-19 years, n=1,304; Malawi: females aged 16-19 years, n=817, males aged 14-19 years, n=1,469; Uganda: females aged 15-19 years, n=1,362, males aged 14-19 years, n=1,787 (Table 3)].

A logistic model was fitted for each sexual act in each country, using STATA (version 10.1) (StataCorp, College Station, TX, USA). Analysis considered factors at the individual, household and community levels; variables significant at the bivariate level were included in the final analysis. Individual- and household-level independent variables were divided into four categories: demographic and economic, parent-adolescent interaction, adolescents’ behaviour and knowledge, and social interaction (Table 1). Demographic and economic factors include age, education level, ethnic group, religious group, participation in a puberty rite, working for pay outside the home, and a weighted household economic index created by assigning point values to household possessions and living conditions and summing the values through a weighted scale. Parent-adolescent interaction includes three variables: parent(s) with whom the adolescent lives, family communication about sex, and a parental monitoring index (0-6) created by summing point values assigned to the frequency at which parents know where the adolescents go, what they do, and who their friends are. Knowledge variables include attendance to sex education and a reproductive health knowledge index created by assigning one point for each correct answer in a series of 37 questions about pregnancy, contraception, HIV/AIDS transmission and treatment, and sexually transmitted infections. Variables relating to adolescents’ behaviour include ever-use of alcohol and an index of media exposure (0-10), in which points were assigned for the frequency of exposure to radio, television, newspaper/magazine, and ever-use of the Internet. Social interaction variables include number of close male and female friends, membership in a group or club, and communication about sex outside of the family.

In the absence of a community survey, individual responses were aggregated at the cluster level to form proxy community measures. Community-level variables (Table 1) were selected based on previous works (11,13-15). Community-level demographic characteristics are represented in this analysis by the proportion of adolescents belonging to the sample's predominant religion, the proportion of adolescent females who are married, and the proportion of adolescents belonging to one or more social group. The potential for future mobility is considered, using the mean of the weighted household economic index. This analysis also considers behaviour and knowledge indicators of adolescents at the community level, represented by the percentage of adolescents who received sexual education, the mean score on reproductive health knowledge index, the mean level of media exposure, and the fraction of adolescents who have ever used alcohol. Parents’ knowledge of adolescents’ life is represented by the mean of the community score on an index measuring how often parents know about the friends and whereabouts of the adolescents and the fraction of adolescents who talk with their family about sex.

## RESULTS

As the sample for analysis was restricted to only adolescents above the median age at first sex by country and gender, mean ages and age-ranges varied across countries and genders, ranging from 16.1 years (Ghanaian males) to 17.3 (Malawian females) (Table 2). Levels of educational attainment varied: the proportion of females who had attained secondary school education or above ranged from 14.4% in Burkina Faso to 68.6% in Ghana. Across all four countries, more females than males were married. Between 33.4% and 59.1% of the young people live with both of their natural parents, and the mean parental monitoring index scores ranged from 3.7 to 4.8. While 59.4% of females in Ghana and 50.2% of females in Uganda had ever received sex education, only 13.8% of females in Burkina Faso and 16.3% of females in Malawi had. Young people reported more friends of the same sex than of the opposite sex in all four countries, and between 10.8% and 39% of the youths belonged to one or more social group or club.

The median ages at first sex in the four countries ranged from 14 among males in Malawi and Uganda to 16 among females in Ghana and Malawi (Table 3). Among young people who were of the same age or older than the median age at first sex, the percentage of those who had engaged in early sex was the highest among females and males in Malawi (18.4% and 19.3% respectively). The proportions were slightly lower in Uganda where 16.8% of females and 14.1% of males had had sex before the median age at first sex. In Burkina Faso, 10.0% of females and 10.5% of males had had early sex. The biggest difference in early initiations of sexual act across genders was seen in Ghana where 15.2% of young women aged 16-19 years had had sex before the age of 16 years compared to 4.3% among young men.

**Table 1.** Definition of individual-, household-, and community-level variables included in the final models of early sex and lifetime sexual partners among adolescents in Burkina Faso, Ghana, Malawi, and Uganda

Individual or household characteristics	Definition
Demographic factors	
Age	Age of respondent at the time of survey
Highest education level	Highest level of schooling attained by adolescent
Ethnic majority	Belong to majority ethnic group of the sample
Religious majority	Belong to majority religious group of the sample
Experienced puberty rite	Ever participated in a puberty or initiation rite
Work for pay	Receive cash for work outside of the home
Weighted household economic index	Index (0-25 in Burkina Faso, 0-28 in others) measuring household living conditions. Index includes: drinking-water source; toilet facility; electricity; household possessions: radio, television, telephone, lamp/lantern in Uganda; refrigerator, sewing machine/cupboard in Uganda (not asked in Burkina Faso); bike, motorcycle, car/truck; type of cooking fuel; floor material; land ownership; home ownership in all countries
Parental interaction	
Lives with parent(s)	Adolescent lives with biological mother, biological father, both, or neither
Parental monitoring index	Index (0-6) refers to level of parental knowledge about adolescents’ life. Created by summing the frequency at which adolescents report that parents know where they go, what they do, and who their friends are.
Family communicates about sex	Adolescents talk about sex within the family
Behaviour and knowledge	
Attended sex education	Adolescent has ever attended sex education
Reproductive health knowledge index	Index (0-36 in Burkina Faso, 0-37 in others) created by adding all correct answers to 37 questions in the areas of pregnancy, contraception, HIV/AIDS transmission and treatment, and STIs
Ever use of alcohol	Ever use of alcohol
Media exposure index	Index (0-10) created to measure the frequency of exposure to radio, television, newspaper/magazine, and ever-use of the Internet
Social interaction	
Male friends	Number of male friends
Female friends	Number of female friends
Talk about sex outside of the family	Ever talk about sex outside of the family
Belongs to a social group	Adolescent belongs to one or more social group or club
Community characteristics (Demographic and future mobility)	
Religious majority	Fraction of adolescents belonging to sample's predominant religion
Married female adolescents	Fraction of female adolescents who have ever been or are currently married
Mean household economic index	Mean score of weighted household economic index
Community social group	Fraction of adolescents belonging to one or more social group or club
Parental interaction	
Talks with family about sex	Fraction of male and female adolescents who report talking with their family about sex
Mean parental monitoring index	Mean score of parental monitoring index
Behaviour and knowledge	
Attended sexual education	Fraction of male and female adolescents who have ever attended sexual education
Mean reproductive health knowledge index	Mean score of reproductive health knowledge index
Mean media exposure index	Mean score of media exposure index
Use of alcohol by adolescents	Fraction of male and female adolescents who have ever used alcohol

The significance and the direction of associations between independent variables and the outcome varied across genders and countries (Table 4). Older adolescents were less likely to report early sex in all countries but Ghana. Females with secondary education in Malawi and Uganda were less likely to report early sex (OR 0.21, 95% CI 0.07-0.64 and OR 0.42, 95% CI 0.18-0.98 respectively) but this effect was not apparent for females in Burkina Faso or Ghana.

Importantly, ethnicity and religious affiliation did not affect early initiation of sexual act in any demographic group, with two exceptions: female adolescents in Burkina Faso were 94% (6%-255%) more likely to report early initiation of sexual act if they did not belong to the majority religious group whereas males in Malawi were 55% (24%-63%) less likely to report early initiation of sexual act if they did not belong to the majority ethnic group. Participating in a puberty rite was not found to be protective against early initiation of sexual act among females, and Malawian males who had participated in a puberty rite were more likely to report early initiation of sexual act compared to Malawian males who had not participated in a puberty rite (OR 1.53, 95% CI 1.12-2.09).

A higher household economic index was associated with lower odds of early sex among females in Burkina Faso (OR 0.92, 95% CI 0.87-.097). In contrast, working for pay was associated with early sex among females in Ghana (OR 1.68, 95% CI 1.02-2.76), males in Malawi (OR 1.62, 95% CI 1.14-2.32), and females in Uganda (OR 1.56, 95% CI 1.01-2.42). Conversely, working outside the home for pay was associated with lower odds of reporting early sex among Ghanaian males (OR 0.28, 95% CI 0.11-0.71).

Parental involvement was also found to affect early age at first sex. While living with both parents was associated with lower odds of early sexual debut only among females in Burkina Faso (OR 0.52, 95% CI 0.35-0.78), higher levels of parental monitoring was associated with lower odds of early sex for females and males in Burkina Faso (OR 0.78, 95% CI 0.70-0.88 and OR 0.78, 95% CI 0.69-0.87 respectively), females and males in Ghana (OR 0.84, 95% CI 0.74-0.97 and OR 0.78, 95% CI 0.65-0.94 respectively), males in Malawi (OR 0.85, 95% CI 0.78-0.92), and females and males in Uganda (OR 0.86, 95% CI 0.76-0.93 and OR 0.81, 95% CI 0.74-0.88 respectively).

**Table 2.** Distribution [% or mean (range)] of individual-, household-, and community-level characteristics among adolescents in Burkina Faso, Ghana, Malawi, and Uganda, 2004

Characteristics	Burkin Faso		Ghana		Malawi		Uganda	
	Females	Males	Females	Males	Females	Males	Females	Males
	(n=1,684)	(n=1,704)	(n=991)	(n=1,304)	(n=817)	(n=1,469)	(n=1,326)	(n=1,787)
Demographic and economic								
Age: mean (range)	16.8 (15,19)	16.7 (15,19)	17.3 (16,19)	16.7 (15,19)	17.3 (16,19)	16.2 (14,19)	16.8 (15,19)	16.1 (14,19)
Highest education level								
None	64.9%	54.1%	10.5%	7.5%	4.2%	2.3%	6.5%	2.9%
Primary	20.8%	27.5%	20.9%	28.9%	65.1%	80.0%	66.2%	72.7%
Secondary or higher	14.4%	18.5%	68.6%	63.6%	30.7%	17.8%	27.4%	24.4%
Belongs to majority ethnic group	51.1%	46.8%	47.8%	46.5%	32.8%	30.1%	20.8%	18.2%
Belongs to majority religious group	57.3%	59.8%	34.1%	30.2%	48.4%	40.6%	41.9%	43.7%
Currently married	23.3%	0.8%	8.9%	0.8%	17.9%	0.8%	18.2%	1.2%
Participated in puberty rite	3.1%	6.1%	7.2%	4.0%	58.6%	35.3%	22.2%	12.3%
Works for pay	15.3%	16.3%	15.6%	20.9%	7.3%	17.1%	13.0%	24.3%
Weighted household economic index	8.8 (2,23)	8.4 (2,22)	13.3 (3,25)	12.4 (3,25)	11.3 (4,23)	11.1 (4,24)	9.9 (2,24)	9.2 (2,24)
Parental interaction								
Lives with parent(s)								
No biological parent	43.2%	23.3%	34.3%	26.1%	43.2%	32.9%	45.1%	29.0%
Mother only	8.1%	9.4%	23.0%	22.0%	19.8%	18.6%	14.5%	18.9%
Father only	5.1%	8.2%	4.4%	9.4%	3.1%	4.9%	7.1%	10.4%
Mother and father	43.6%	59.1%	38.3%	42.5%	33.9%	43.6%	33.4%	41.8%
Parental monitoring index	4.7 (0,6)	4.1 (0,6)	4.8 (0,6)	4.3 (0,6)	4.4 (0,6)	3.7 (0,6)	4.4 (0,6)	3.8 (0,6)
Talks with family about sex	22.5%	18.2%	53.6%	33.8%	43.5%	35.2%	52.1%	29.8%
Behaviour and knowledge								
Attended sex education	13.8%	18.2%	59.4%	44.8%	16.3%	26.3%	50.2%	41.2%
Reproductive health knowledge index	16.6 (0,32)	16.5 (0,34)	23.1%	22.8 (0,36)	24.2 (0,36)	22.9 (0,35)	23.4 (0,36)	22.8 (0,34)
Ever use of alcohol	36.4%	40.2%	26.3%	31.6%	14.1%	22.2%	30.6%	40.5%
Media exposure index	2.4 (0,9)	3.0 (0,10)	4.5 (0,10)	4.7 (0,10)	3.1 (0,10)	3.7 (0,10)	3.0 (0,10)	3.7 (0,10)
Social interaction								
Number of close male friends	0.7 (0,20)	3.4 (0,22)	1.8 (0,50)	4.1 (0,45)	1.1 (0,11)	3.7 (0,25)	3.0 (0,10)	6.2 (0,50)
Number of close female friends	2.4 (0,25)	1.0 (0,15)	2.8 (0,30)	1.9 (0,20)	2.8 (0,12)	1.8 (0,20)	1.8 (0,20)	2.5 (0,50)
Talks about sex outside of family	34.4%	51.1%	59.2%	55.3%	41.4%	51.6%	52.0%	57.4%
Belongs to social group	10.8%	11.0%	39.0%	24.1%	26.1%	30.3%	22.2%	17.8%
Community-level characteristics: mean (range)								
Adolescents belonging to majority religious group	0.57 (0,1)	0.57 (0,1)	0.34 (0,0.86)	0.34 (0,0.86)	0.43 (0,1)	0.43 (0,1)	0.43 (0,1)	0.43 (0,1)
Married adolescent women	0.14 (0,0.75)	0.14 (0,0.75)	0.06 (0,1)	0.06 (0,1)	0.11 (0,0.57)	0.11 (0,0.57)	0.12 (0,10)	0.12 (0,10)
Household economic index	0.19 (-4.39,8.23)	0.19 (-4.39,8.23)	0.25 (-5.64,7.29)	0.25 (-5.64,7.29)	0.7 (-5.34,6.04)	0.7 (-5.34,6.04)	0.16 (-4.77,9.05)	0.16 (-4.77,9.05)
Adolescent belongs to social group	0.09 (0,0.41)	0.09 (0,0.41)	0.28 (0,0.73)	0.28 (0,0.73)	0.27 (0,0.65)	0.27 (0,0.65)	0.19 (0,0.6)	0.19 (0,0.6)
Parental monitoring index	4.56 (3,5.65)	4.56 (3,5.65)	4.71 (3.44,5.86)	4.71 (3.44,5.86)	4.16 (2.07,5.78)	4.16 (2.07,5.78)	4.36 (2.31,5.64)	4.36 (2.31,5.64)
Adolescent talks with family about sex	0.16 (0,0.45)	0.16 (0,0.45)	0.41 (0,0.91)	0.41 (0,0.91)	0.34 (0,0.79)	0.34 (0,0.79)	0.38 (0,0.79)	0.38 (0,0.79)
Ever attended sexual education	0.15 (0,0.61)	0.15 (0,0.61)	0.46 (0,0.95)	0.46 (0,0.95)	0.19 (0,0.54)	0.19 (0,0.54)	0.40 (0,0.81)	0.40 (0,0.81)
Reproductive health knowledge index	16.42 (7.56,22.91)	16.42 (7.56,22.91)	20.90 (4.83,27.71)	20.90 (4.83,27.71)	3.09 (12.07,26.83)	3.09 (12.07,26.83)	20.64 (10.57,26.68)	20.64 (10.57, 26.68)
Media exposure index	2.56 (0.37,5.12)	2.56 (0.37,5.12)	4.46 (0.58, 7.2)	4.46 (0.58,7.2)	1.12 (1,7)	1.12 (1,7)	3.23 (0.03,7.27)	3.23 (0.03,7.27)
Adolescent ever had alcohol	0.37 (0,0.97)	0.37 (0,0.97)	0.24 (0,0.75)	0.24 (0,0.75)	0.16 (0,0.5)	0.16 (0,0.5)	0.33 (0.03,0.87)	0.33 (0.03,0.87)

**Table 3.** Sample-size, median age at first sex, and prevalence of early initiation of sexual act among male and female adolescents in Burkina Faso, Ghana, Malawi, and Uganda, 2004

Age at first sex	Burkina Faso		Ghana		Malawi		Uganda	
	Females (n=1,684)	Males (n=1,704)	Females (n=991)	Males (n=1,304)	Females (n-817)	Males (n=1,469)	Females (n=1,362)	Males (n=1,787)
Median age at first sex	15	15	16	15	16	14	15	14
Early age at first sex*	10.0%	10.5%	15.2%	4.3%	18.4%	19.3%	16.8%	14.1%

*Percentage of adolescents at or over the median age at first sex, who reported sex before the median age

Adolescents with higher levels of reproductive health knowledge were 7%-13% (3%-19%) more likely to report early sex in all demographic groups, except for females in Burkina Faso and males in Ghana.

Ever-use of alcohol was also associated with early sexual debut. Males in Burkina Faso (OR 1.85, 95% CI 1.18-2.91), females and males in Ghana (OR 1.89, 95% CI 1.19-3.01 and OR 2.31, 95% CI 1.19-4.49 respectively), and females and males in Uganda (OR 1.90, 95% CI 1.33-2.74 and OR 1.73, 95% CI 1.25-2.39 respectively) who had ever consumed alcohol were more likely to report early sex compared to adolescents who reported never having used alcohol. Exposure to media had mixed associations with early sex. Females in Burkina Faso with greater media exposure were more likely to have early sex (OR 1.14, 95% CI 1.01-1.30) while females with higher levels of media exposure in Malawi and Uganda were less likely to report early initiation of sexual act (OR 0.84, 95% CI 0.74-0.95 and 0.83, 95% CI 0.74-0.92 respectively).

Young people who had more friends of the opposite sex were more likely to report early sex. Females who reported having more close male friends were more likely to have had early sex in Malawi (OR 1.19, 95% CI 1.05-1.36), and males who reported having more close female friends were more likely to have initiated sex before the median age at first sex in Burkina Faso (OR 1.21, 95% CI 1.09-1.34) and Ghana (OR 1.18, 95% CI 1.04-1.33). Having more close friends of the same sex, however, was protective of early sex. Females who reported more close female friends were less likely to have had early sex in Malawi (OR 0.83, 95% CI 0.71-0.96). Talking with people outside of the family about sex was associated with early sex only among males in Burkina Faso (OR 1.89, 95% CI 1.25-2.86), and males in Burkina Faso, who reported belonging to a social group or club, were more likely to report having had early sex (OR 1.87, 95% CI 1.15-3.03).

Associations with community-level factors also varied across genders and countries. Living in a community with a larger percentage of adolescents belonging to the predominant religious group was associated with lower odds of early sex among females and males in Malawi (OR 0.30, 95% CI 0.11-0.84 and OR 0.46, 95% CI 0.22-0.96). Adolescent females who lived in a community with more married adolescents were more likely to have early sex in Burkina Faso (OR 5.76, 95% CI 1.34-24.85), Ghana (OR 18.41, 95% CI 2.24-146.90), and Uganda (OR 11.03, 95% CI 3.38-35.94). Males in Uganda, who lived in a community with more married females, are also more likely to have early sex (OR 3.44, 95% CI 1.04-11.37). Living in a community with a higher mean economic level was associated with lower odds of reporting early initiation of sexual act only for Ugandan males (OR 0.85, 95% CI 0.77-0.94). Adolescents who lived in communities with a larger percentage of young people involved in social groups were less likely to report having early sex in Malawi (OR 0.12, 95% CI 0.02-0.70 among females and OR 0.21, 95% CI 0.06-0.74 among males) but were more likely to report having early sex in Ghana (OR 5.32, 95% CI 1.09-26.06 among females).

Higher levels of communication about sex with family members at the community level were associated with early sex only for males in Malawi (OR 4.80, 95% CI 1.50-15.31). Ugandan females (OR 1.15, 95% CI 1.03-1.28) and males (OR 1.12, 95% CI 1.02-1.23) living in communities with higher mean levels of reproductive health knowledge were more likely to report having had early sex. Higher media exposure at the community level were associated with lower odds of early sex for only females in Burkina Faso (OR 0.67). Finally, living in a community with a larger percentage of adolescents who had ever tried alcohol was associated with lower odds of early sex among males in Burkina Faso (OR 0.11, 95% CI 0.03-0.48), females in Ghana (OR 0.17, 95% CI 0.03-0.89), males in Malawi (OR 0.17, 95% CI 0.04-0.73), and both females and males in Uganda (OR 0.21, 95% CI 0.06-0.69 and OR 0.20, 95% CI 0.06-0.69 respectively).

**Table 4.** Multivariate model for early sex among females and males in Burkina Faso, Ghana, Malawi, and Uganda, 2004

Characteristics	Burkin Faso		Ghana		Malawi		Uganda	
	Female	Male	Female	Male	Female	Male	Female	Male
	(n=1,684)	(n=1,704)	(n=991)	(n=1,304)	(n=817)	(n=1,469)	(n=1,362)	(n=1,787)
Current age of respondent	***0.87***	***0.77***	1.01	1.05	0.86	0.86	0.81	0.77
	***(0.76-0.99)***	***(0.68-0.88)***	(0.84-1.21)	(0.82-1.35)	(0.70-1.06)	(0.78-0.95)	(0.72-0.92)	(0.69-0.85)
Highest educational level (none)								
Primary	0.77	1.16	1.80	1.20	0.40	1.45	0.90	0.63
	(0.46-1.28)	(0.77-1.77)	(0.83-3.89)	(0.33-4.33)	(0.14-1.08)	(0.53-3.92)	(0.45-1.84)	(0.22-1.80)
Middle (Ghana)	n/a	n/a	1.59 (0.74-3.43)	0.98 (0.25-3.80)	n/a	n/a	n/a	n/a
Secondary or higher	0.54	1.15	0.46	0.72	***0.21***	1.24	***0.42***	0.56
	(0.25-1.15)	(0.64-2.08)	(0.16-1.35)	(0.14-3.80)	***(0.07-0.64)***	(0.42-3.69)	***(0.18-0.98)***	(0.18-1.74)
Work	1.52	1.19	***1.68***	***0.28***	0.68	***1.62***	***1.56***	0.82
	(0.96-2.39)	(0.75-1.87)	***(1.02-2.76)***	***(0.11-0.71)***	(0.31-1.50)	***(1.14-2.32)***	***(1.01-2.42)***	(0.56-1.18)
Ethnic majority (no)	0.85	1.32	0.95	0.60	***0.45***	1.29	0.84	0.96
	(0.58-1.26)	(0.92-1.89)	(0.60-1.50)	(0.29-1.23)	***(0.27-0.76)***	(0.92-1.80)	(0.52-1.36)	(0.61-1.50)
Religious majority (no)	***1.94***	1.41	1.01	1.23	1.06	0.76	0.82	1.02
	***(1.06-3.55)***	(0.83-2.41)	(0.63-1.61)	(0.59-2.55)	(0.68-1.67)	(0.55-1.07)	(0.57-1.20)	(0.71-1.45)
Lives with parent (neither)								
Mother only	0.52	1.17	0.87	0.87	0.88	1.09	0.93	1.19
	(0.25-1.09)	(0.63-2.16)	(0.52-1.47)	(0.36-2.11)	(0.52-1.49)	(0.73-1.63)	(0.57-1.50)	(0.78-1.82)
Father only	***0.29***	0.59	1.39	0.62	0.54	1.27	0.55	1.31
	***(0.09-0.98)***	(0.27-1.26)	(0.57-3.35)	(0.18-2.11)	(0.11-2.56)	(0.67-2.44)	(0.27-1.12)	(0.78-2.22)
Both	***0.52***	0.78	0.66	1.05	0.62	0.90	0.66	0.94
	***(0.35-0.78)***	(0.52-1.16)	(0.40-1.06)	(0.49-2.23)	(0.38-1.02)	(0.64-1.25)	(0.44-1.00)	(0.65-1.38)
Media exposure index	***1.14***	0.99	0.98	0.96	***0.84***	0.99	***0.83***	1.00
	***(1.01-1.30)***	(0.88-1.12)	(0.87-1.10)	(0.78-1.18)	***(0.74-0.95)***	(0.91-1.07)	***(0.74-0.92)***	(0.91-1.10)
Belongs to social group	0.94	***1.87***	0.76	0.50	0.65	0.96	1.07	1.16
	(0.51-1.74)	***(1.15-3.03)***	(0.48-1.21)	(0.21-1.17)	(0.38-1.11)	(0.69-1.34)	(0.69-1.66)	(0.78-1.73)
RH knowledge index	1.03	***1.07***	***1.07***	1.03	***1.13***	***1.07***	***1.08***	***1.09***
	(0.99-1.06)	***(1.03-1.11)***	***(1.03-1.12)***	(0.97-1.10)	***(1.08-1.19)***	***(1.04-1.11)***	***(1.04-1.12)***	***(1.05-1.13)***
Participated in puberty rite	1.62	0.87	0.78	1.89	1.48	***1.53***	1.31	1.49
	(0.68-3.87)	(0.45-1.67)	(0.37-1.65)	(0.55-6.51)	(0.93-2.35)	***(1.12-2.09)***	(0.84-2.05)	(0.98-2.28)
Person outside of the family talks about sexual matters	1.12	***1.89***	1.01	1.63	0.75	1.25	1.14	1.24
	(0.76-1.67)	***(1.25-2.86)***	(0.64-1.60)	(0.82-3.23)	(0.48-1.16)	(0.91-1.72)	(0.81-1.61)	(0.88-1.73)
Number of close female friends	1.04	***1.21***	0.99	***1.18***	***0.83***	1.05	1.00	***1.05***
	(0.95-1.14)	***(1.09-1.34)***	(0.90-1.07)	***(1.04-1.33)***	***(0.71-0.96)***	(0.97-1.13)	(0.95-1.05)	***(1.01-1.10)***
Number of close male friends	1.03	1.00	1.01	0.98	***1.19***	0.95	1.03	1.00
	(0.90-1.18)	(0.93-1.07)	(0.94-1.08)	(0.90-1.07)	***(1.05-1.36)***	(0.89-1.01)	(0.97-1.10)	(0.97-1.02)
Parents' knowledge	***0.78***	***0.78***	***0.84***	***0.78***	0.90	***0.85***	***0.86***	***0.81***
	***(0.70-0.88)***	***(0.69-0.87)***	***(0.74-0.97)***	***(0.65-0.94)***	(0.80-1.01)	***(0.78-0.92)***	***(0.79-0.93)***	***(0.74-0.88)***
Ever drank alcohol	1.46	***1.85***	***1.89***	***2.31***	1.83	1.39	***1.90***	***1.73***
	(0.86-2.47)	***(1.18-2.91)***	***(1.19-3.01)***	***(1.19-4.49)***	(0.99-3.39)	(0.98-1.98)	***(1.33-2.74)***	***(1.25-2.39)***
Weighted economic index	***0.92***	1.00	0.97	1.03	1.01	0.98	1.02	1.02
	***(0.87-0.97)***	(0.95-1.05)	(0.92-1.02)	(0.96-1.12)	(0.96-1.05)	(0.95-1.02)	(0.97-1.06)	(0.98-1.07)
Community economic index	1.11	0.95	1.02	1.07	1.08	1.08	1.01	***0.85***
	(0.98-1.25)	(0.85-1.06)	(0.92-1.14)	(0.90-1.26)	(0.96-1.22)	(0.99-1.17)	(0.92-1.11)	***(0.77-0.94)***
Community religion	1.75	0.42	0.61	1.45	***0.30***	***0.46***	0.97	1.03
	(0.44-6.97)	(0.12-1.40)	(0.19-1.93)	(0.26-8.24)	***(0.11-0.84)***	***(0.22-0.96)***	(0.43-2.21)	(0.47-2.25)
Community social group	0.89	2.87	***5.32***	0.97	***0.12***	***0.21***	0.36	1.08
	(0.07-11.76)	(0.25-33.34)	***(1.09-26.06)***	(0.07-13.39)	***(0.02-0.70)***	***(0.06-0.74)***	(0.07-1.90)	(0.26-4.54)
Community RH knowledge	0.99	1.00	0.98	0.98	0.92	0.99	***1.15***	***1.12***
	(0.90-1.10)	(0.90-1.11)	(0.89-1.09)	(0.83-1.15)	(0.83-1.02)	(0.91-1.06)	***(1.03-1.28)***	***(1.02-1.23)***
Community media exposure	***0.67***	0.74	0.83	0.75	1.16	1.06	0.87	0.85
	***(0.49-0.91)***	(0.55-1.01)	(0.61-1.12)	(0.46-1.20)	(0.85-1.58)	(0.85-1.33)	(0.69-1.09)	(0.67-1.06)
Community talks with family about sex	1.39	1.41	1.62	2.06	5.19	***4.80***	1.57	0.68
	(0.14-13.57)	(0.15-13.19)	(0.48-5.47)	(0.36-11.83)	(0.93-28.84)	***(1.50-15.31)***	(0.43-5.70)	(0.20-2.28)
Community marriage	***5.76***	2.78	***18.14***	1.69	1.62	0.53	***11.03***	***3.44***
	***(1.34-24.85)***	(0.54-14.31)	***(2.24-146.90)***	(0.18-16.17)	(0.25-10.44)	(0.13-2.11)	***(3.38-35.94)***	***(1.04-11.37)***
Community alcohol	1.04	***0.11***	***0.17***	2.94	3.69	***0.17***	***0.21***	***0.20***
	(0.22-5.02)	***(0.03-0.48)***	***(0.03-0.89)***	(0.30-28.59)	(0.42-32.41)	***(0.04-0.73)***	***(0.06-0.75)***	***(0.06-0.69)***

Early sex defined as sex before the median age at first sex; Values are OR (95% CI); Significant results (p<0.05) are shown in ***bold italic***

## DISCUSSION

While the results of this study support the previously-found associations between early sexual debut and individual demographic and economic factors among adolescents, the findings stress the context-specific influence of communities and places on adolescent sexual health in sub-Saharan Africa. For example, the protective effects of education (31,48) and wealth (10) against early sex have been established. However, the associations that community levels of wealth and community martial practices have with early initiation of sexual act are important new findings. Higher community levels of wealth were found to be protective of early age at first sex, which supports Billy *et al.*'s theory that young people who can identify pathways to future success will make less risky sexual decisions because the consequences are greater (11). The proportion of married adolescent females in a community was also associated with early sex for females in three countries (Burkina Faso, Malawi, and Uganda) but, for males in only one country (Uganda), community levels of early marriage impact early age at first sex for adolescent females comparatively more than adolescent males. This may reflect variations in expected sexual and marital behaviours across genders: a large percentage of adolescent females who are married may reflect community norms and expectations that females marry and begin sexual activity at an earlier age than males in order to conform to traditional notions of feminine identity.

Similarly, the relationship between individual use of alcohol and early sex confirms findings from previous studies (25,31,36). However, it is surprising that, adjusting for use of alcohol at the individual level, higher use of alcohol at the community level among adolescents was associated with lower odds of early sex. A possible explanation for this is that places where adolescents have more access to alcohol may also be wealthier and, thus, provide more opportunities for upward mobility, which would increase the ‘opportunity cost’ of risky sexual behaviour as suggested by Billy *et al.* (11). This novel and unusual result requires further investigation to understand the contrasting findings between use of alcohol at the individual and community levels.

This study also confirms previous findings of the protective role of the parent-adolescent relationship in sexual behaviour (1,9,23,31,37). Increased parental knowledge of an adolescent's activities, whereabouts, and friends was associated with 14-22% decrease in odds of early initiation of sexual act in nearly all genders/countries. Families in which the parent(s) are more involved with and knowledgeable about an adolescent's life may be structured as to provide fewer opportunities for adolescents to engage in sex, and parental involvement and interest in adolescents’ life likely reduces desire for risk-taking among adolescents. Additionally, adolescents with involved parents may be more aware of the potential consequences of early initiation of sexual act. However, for Malawian males, living in a community where more parents and families discuss sex with their children increased the odds of early sex. Clearly, parent-child discussions around sex are context-specific, and it is possible that discussions in Malawi contain more information on safe sex rather than abstinence. Given the high HIV prevalence in Malawi, it is possible that communities with high levels of early adolescent sexual activity may have been targeted for sexual health interventions and messaging which encourage parents to talk to their children about safer sex. The results may reflect endogeneity or targeting of programmatic efforts to high-risk communities, thus creating simultaneous high levels of risky behaviour and parent-child interaction.

At the individual level, the results confirm previous findings that associations between social group membership and risky sex vary by country (31,38). However, at the community level, higher levels of involvement in social groups are protective of early age at first sex more in two contexts—Ghana and Malawi—suggesting that communities with higher levels of adolescents’ involvement in groups may be supportive of delayed sexual debut by having social norms that support adolescents as valuable members of their community or providing alternative social activities for adolescents. Our finding that having more friends of the opposite sex is associated with early age at first sex and that having more friends of the same sex is associated with the inverse supports the finding of Kumi-Kyerme *et al.* (5) in Ghana. While this result may be misclassified by adolescents reporting sexual partners as close friends, adolescents who have more friends of the opposite sex may, indeed, have more opportunities for sex and, thus, have earlier ages of sexual debut. Accordingly, friends of the same sex may provide the adolescent with support systems that encourage later sexual debut or provide opportunities for social interactions that do not include sexual behaviour.

The need to consider community and context may be further evidenced by the lack of co-directionality in the association between working outside the home and early age at first sex—working outside the home was associated with lower odds of reporting early sex for Ghanaian males but higher odds of reporting early first sex for Malawian males. Working outside the home was also associated with higher odds of early sexual debut for females in both Ghana and Malawi. It is possible that, in some places, working outside the home increases the social interaction of an adolescent and provides more opportunities for sex. Working may also increase the economic power of a young person, potentially allowing him or her to negotiate or maintain transactional sexual relationships. In other contexts, however, working outside the home may reduce time for socialization and, thus, reduce opportunities for adolescents to engage in early sex. Although previous studies have shown a protective relationship between reproductive health knowledge and risky sexual behaviour (31), the present findings illustrate the converse at both individual level and the community level. However, due to the cross-sectional nature of the data, it cannot be determined if reproductive health knowledge preceded initiation of sexual act. It is possible that adolescents reported increased knowledge of reproductive health gained primarily through sexual experiences. This function may also be at work in communities. If higher overall knowledge of reproductive health is linked to higher levels of sexual activity as suggested by these results, this would, in turn, likely correlate to higher levels of reproductive health knowledge in a community. Additionally, this result may reflect endogeneity, wherein information and interventions are targeted towards communities in need, i.e. communities that are known to have high levels of sexually-active adolescents would receive reproductive health information from programmatic efforts, the results of which may not yet have been translated into behavioural change.

Finally, this analysis found several areas in which further research is needed. For example, the association between participation in a puberty rite and increased odds of early sexual debut is interesting and relatively undocumented. It is possible that puberty rites are found in places with more traditional communities where earlier marriage and earlier sexual debuts are common, or perhaps that adolescents are either allowed or expected to engage in intercourse after passing a puberty rite in some communities. Further research focusing on the types of puberty rites and the contextual ramifications of different puberty rites is needed to fully understand this association.

### Strengths and limitations

This analysis has several limitations. First, the data are based on self-reported sexual behaviour. Misreporting of sexual behaviour is possible, particularly among young populations (49,50). However, the surveys used in this analysis are unique in both breadth and depth of information gathered and are the only nationally-representative surveys in these four countries that include young people aged 12-19 years. Thus, the importance of the information gained from these analyses outweighs the potential for bias. The cross-sectional nature of the surveys also prevents further understanding of the causal relationships surrounding sexual behaviour among adolescents. Finally, individual responses by adolescents were used in creating community-level variables because no community-level data were available. Therefore, the analyses were unable to consider community factors, such as the characteristics of the adult population, community economic development, and availability of health services. However, the results presented here form a strong foundation for the development of further research that would include such place-related factors.

### Conclusions

The central finding from this study is that place-specific characteristics of different communities have dissimilar effects on adolescents’ sexual health in the same way that individual demographic characteristics do. Particularly, the findings from this analysis illustrate the importance of the place in which young people live in shaping sexual behaviour. In addition, the differences in risk and protective factors across genders and countries underscore the need for a context-specific understanding of the factors that influence sexual behaviour of adolescents and draw critical attention to universalist strategies. Given the high costs of early sexual debut, including HIV and unintended pregnancy, there is an urgent need for additional novel research methods, such as the ones presented here. Community-level factors that are associated with sexual behaviour should be harnessed in the development of place-specific programmes to promote sexual health of adolescents.
